# Assessing the influence of lived-experience experts on healthcare providers in a virtual community of practice: a qualitative study

**DOI:** 10.3389/frhs.2025.1562651

**Published:** 2025-06-27

**Authors:** Stephanie Sargent Weaver, Monique Carry, Jeanne Bertolli, Job Godino, Bruce Struminger, Douglas Taren, John D. Scott, Sydney P. Sharp, Jane Samaniego, Donna R. Bean, Anindita Issa, Jin-Mann S. Lin, Elizabeth R. Unger, Christian B. Ramers

**Affiliations:** ^1^ Centers for Disease Control and Prevention, National Center for Emerging and Zoonotic Infectious Diseases, Chronic Viral Diseases Branch, Atlanta, GA, United States; ^2^ Centers for Disease Control and Prevention, Global Health Center, HIV Prevention Branch, Atlanta, GA, United States; ^3^ Herbert Wertheim School of Public Health and Human Longevity Science, University of California, San Diego, La Jolla, CA, United States; ^4^ Laura Rodriguez Research Institute, Family Health Centers of San Diego, San Diego, CA, United States; ^5^ Department of Internal Medicine, University of New Mexico, Albuquerque, NM, United States; ^6^ Department of Pediatrics, Nutrition Section, University of Colorado School of Medicine, Aurora, CO, United States; ^7^ Allergy and Infectious Diseases, University of Washington, Seattle, WA, United States; ^8^ PHI/CDC Global Health Fellowship Program, Public Health Institute, Oakland, CA, United States

**Keywords:** Long COVID, myalgic encephalomyelitis/chronic fatigue syndrome (ME/CFS), post-acute infection syndromes (PAIS), Extension for Community Healthcare Outcomes (ECHO) learning model, lived-experience experts, qualitative evaluation

## Abstract

Long COVID, myalgic encephalomyelitis/chronic fatigue syndrome (ME/CFS), and other poorly understood post-acute infection syndromes (PAIS) can present with unexplained symptoms or conditions that may be misunderstood by healthcare providers, causing delays in diagnosis and care. To address these issues, the Centers for Disease Control and Prevention (CDC) funded the Long COVID and Fatiguing Illness Recovery Program (LC&FIRP), initiated as a pilot project to assess whether providing tele-mentoring and other online education for primary care providers could help them improve the quality of life and support the recovery of their patients with these conditions. The LC&FIRP multi-disciplinary team-based care approach is built on the Extension for Community Healthcare Outcomes (ECHO) learning model, which is an evidence-based virtual learning framework developed by the University of New Mexico and designed to disseminate and implement best practices, especially in under-resourced areas. A distinctive feature of LC&FIRP was the inclusion of lived-experience experts. To explore the influence of lived-experience experts on the care patients received, we collected the educational recommendations provided by the lived-experience experts during webinar sessions (January 2022—March 2024) and grouped these by themes. The major themes that emerged included validation of patients’ illness experience; attitudes and beliefs about Long COVID, ME/CFS, and PAIS; understanding patients’ challenges and communicating with empathy; navigating referrals; recognizing and supporting disability; and supporting self-care. Investigators also interviewed patients of the Family Health Centers of San Diego (FHCSD) about their experiences receiving care from participating primary care providers and employed content analysis methods to code interview transcripts to identify themes among patients’ perspectives. Positive comments from the patients about topics emphasized by the lived-experience experts provided evidence of providers’ uptake and application of the experts’ recommendations and support the value of involving lived-experience experts in medical education to improve health services.

## Introduction

Long COVID is a chronic condition that follows a SARS-CoV-2 infection and lasts at least 3 months; it is considered one of several poorly understood chronic health problems associated with infection (1). People with Long COVID experience a wide range of symptoms and conditions, some of which are similar to those reported by individuals with myalgic encephalomyelitis/chronic fatigue syndrome (ME/CFS) and other post-acute infection syndromes (PAIS). These currently unexplained symptoms or conditions may be misunderstood by healthcare providers, which can result in a delay in diagnosis and receipt of appropriate care or treatment (2, 3). In 2022, an estimated 3.5% of U.S. adults were currently experiencing Long COVID (4), and in 2021–2022, 1.3% of adults had ME/CFS (5).

Long COVID, ME/CFS, and other poorly understood PAIS are challenging to recognize and treat because there are currently no diagnostic biomarkers or approved pharmacologic therapies. Care and treatment are focused on the management of symptoms to reduce their impact, counseling on pacing, and other energy management strategies. These strategies are ideally tailored to the individual patient because clinical profiles are so variable (6–8). The number of healthcare providers equipped to care for patients with Long COVID and similar post-acute infection syndromes is unknown, but likely severely inadequate to the clinical need (9). Long COVID spurred the establishment of Long COVID multi-disciplinary clinics, which sometimes, but not always, accept patients with symptoms overlapping with Long COVID and without documentation of a SARS-CoV-2 infection. Anecdotal reports indicate that the number of Long COVID clinics remains insufficient to address the demand (10). Similarly, the small number of ME/CFS specialists practicing in the U.S. is overwhelmed by demand for their services (3, 11).

To address these workforce limitations, the CDC-funded Long COVID and Fatiguing Illness Recovery Program (LC&FIRP) was initiated as a pilot project to assess whether providing tele-mentoring and other online education for primary care providers could help them improve the quality of life and support the recovery of their patients with Long COVID, ME/CFS, and PAIS. The LC&FIRP multi-disciplinary team-based care approach is built on the Extension for Community Healthcare Outcomes (ECHO) learning model (12). The ECHO continuing education model is an evidence-based virtual learning framework developed by the University of New Mexico and designed to disseminate and implement best practices, especially in under-resourced areas. The participants engage in a case-based collaborative tele-mentoring approach by recognized experts in their specialties, and multi-disciplinary peer-to-peer sharing of emerging promising practices, as well as demonstrating support, guidance, and providing feedback on attendees' actual patient cases. In this instance, tele-mentoring is defined as “a relationship, facilitated by telecommunication technology, in which an expert (mentor) provides guidance to a less experienced learner (mentee) from a remote location” (13). Previous research has demonstrated the utility of applying the ECHO model as an effective way to increase workforce capacity to treat Hepatitis C infection in underserved communities (14), and this model is being implemented nationally and internationally for the clinical management of a wide variety of other acute and chronic conditions.

The LC&FIRP program applies the ECHO distance learning model to the management of complex PAIS like Long COVID and ME/CFS. The LC&FIRP collaborators include the Family Health Centers of San Diego, a federally qualified health center network, and its academic partners at the University of New Mexico ECHO Institute, the University of Colorado School of Medicine, and the University of Washington School of Medicine, with support from AtaHealth Strategies. Providers participating in the continuing professional development program attend weekly tele-mentoring sessions with experts in relevant medical disciplines, monthly webinars, and on-demand short courses on a variety of topics that help educate them on current research as well as tips for clinical management of patients with PAIS. A distinctive feature of the LC&FIRP adaptation of the ECHO model is the inclusion of lived-experience experts to provide a perspective on the illness and medical care experiences of people with PAIS. For this paper, the term “people with lived-experience” includes people with living (current) experience or those who are primary caregivers of persons with direct lived experience. Lived-experience experts were integrated with medical specialists in all LC&FIRP educational interventions for primary care providers. This manuscript explores the influence of lived-experience experts on the care patients received from providers exposed to LC&FIRP educational interventions in the first 9 months of the program. It describes how key messages were underscored and elaborated on in the project's second and third years.

## Methods

### Collection of lived-experience experts' input

Lived-experience experts were either persons living with Long COVID, ME/CFS, or another similar PAIS (7) or a caregiver of a person living with one of these syndromes. Nine lived-experience experts participated in the LC&FIRP webinars from January to September 2022; eight had Long COVID or ME/CFS or both, and one was a caregiver for a relative with ME/CFS. Five of the lived-experience experts were affiliated with a patient advocacy organization, two were health care providers, one was a former community health worker/case manager, and one was a medical student. Lived-experience experts were recruited through the LC&FIRP faculty mentors and groups advocating for patients living with PAIS. Two researchers from the CDC independently reviewed recordings of LC&FIRP webinars that were held between January 2022 and March 2024. We collected educational contributions of lived-experience experts and met to review comments and quotations before grouping them by theme.

### Collection of patients' perspectives

We employed an interpretivist, qualitative, descriptive design using semi-structured, in-depth interviews to explore patients' experiences with LC&FIRP and to assess the perceived implementation of recommendations articulated by lived-experience experts during clinician education activities. A content-analytic approach—specifically, conventional content analysis—guided coding and theme development. Below we provide information about the methods that aligns with the COREQ checklist (Supplementary Table S1) (15).

Two female researchers, coauthors DRB and MC, conducted the participant interviews to minimize rater bias. One interviewer was proficient in English, while the other was bilingual in English and Spanish. Both researchers possessed expertise in qualitative evaluation and data collection, independent of FHCSD. Prior to this evaluation, none of the study participants were acquainted with the interviewers. During the interviews, participants were informed of the interviewers' names and roles on the evaluation team, and the voluntary nature of their participation. The interviewers did not disclose their titles or professional backgrounds unless directly requested. At the time of the interviews, DRB served as a Public Health Specialist in the Public Health Institute (PHI)/CDC Global Health Fellowship Program, while MC was a Behavioral Scientist and Senior Communication Advisor in the Division of Global HIV and TB at the CDC's Global Health Center. Both were deployed to the CDC's COVID-19 Emergency Response.

DRB is trained as both a nurse and an epidemiologist, holding a registered nurse (RN) degree and a master's degree in public health. At the time of this evaluation, she had 10 years of clinical nursing experience and 15 years in public health. She had previously applied her graduate training in qualitative methods to public health projects and in response to infectious disease outbreaks. MC holds both master's and doctoral degrees in sociology. She completed doctoral training in qualitative research. At the start of this evaluation, she had 11 years of experience in behavioral science and health communications roles at the CDC. Her research and evaluation work includes qualitative methods for examining policies, systems, and initiatives aimed at enhancing population health. Both researchers completed study-specific training covering reflexivity, trauma-informed interviewing, and cultural humility.

FHCSD staff employed purposeful sampling, aiming to capture variation in age, sex, language (English/Spanish), and illness severity among patients receiving care from LC&FIRP clinicians, with a goal of achieving a total sample size of 16 to 24 participants. Patients at the Family Health Centers of San Diego were recruited for semi-structured qualitative interviews if they met the inclusion criteria for the evaluation, as described below. Twenty-two patients enrolled and participated in the in-depth interviews.

Patients were eligible to participate if they were (1) 18 years or older; (2) able to speak and read fluently in English or Spanish; (3) were receiving care from an LC&FIRP healthcare provider; and (4) consented to have their health information shared for research (FHCSD Broad Consent). Staff at FHCSD identified LC&FIRP participants from clinic records who met the inclusion criteria and were willing to be interviewed. Upon identification, a program coordinator at FHCSD contacted patients by telephone or email, shared the details of this proposed evaluation and inquired about their interest and willingness to be interviewed. Once the patient agreed to participate, the program coordinator at FHCSD scheduled an interview date and time. At that time, the program coordinator at FHCSD sent a Zoom meeting invite with a meeting link and dial-in number to the participant corresponding to their agreed-upon interview date and time. Participants received a reminder text or email twenty-four hours before their scheduled interview.

All in-depth interviews were conducted remotely via Zoom with end-to-end encryption. Participants joined from their private homes, and no non-participants were present. The interview format (i.e., videoconferencing or audio-only) was determined based on participants' preference. The in-depth interviews lasted approximately 30–75 min (Mean = 52 min). If the respondent did not agree to be recorded, the interviewer took real-time field notes that captured nonverbal cues and context. No repeat interviews were conducted.

Before the interviews, the program coordinator at FHCSD sent a copy of the evaluation description and informed consent to participants via email or text before their interview. This allowed participants sufficient time to read the documents and identify any concerns. The in-depth semi-structured patient interviews were conducted over eight weeks in October and November 2022, roughly nine months into the program. Each interview was assigned a unique identification code that was applied to the data.

Topics for a semi-structured patient interview guide were elicited from the project implementation team, and covered two main domains: program assessment, and post-intervention effects. The interview guide was piloted with two patients and refined before data collection. Interview questions, listed in Supplementary Table S2, were designed to elicit aspects of care that were salient to patients and raised spontaneously by them, rather than explicitly asking them to comment on aspects of care that lived-experience experts addressed. In-depth interviews were conducted in an open-ended, conversational manner, with the questions used to guide discussion rather than being asked verbatim.

Interviewers used written reflexive journals to bracket professional assumptions about Long COVID, ME/CFS, and PAIS and minimize confirmatory bias. Weekly debriefs were held with the three-member evaluation team to discuss emerging impressions and mitigate individual bias. Impressions (e.g., similarities, differences, new information) about topics arising during the in-depth interviews were discussed by the team and written in summary notes. At that time, changes, if needed, were made to the interview guides to capture new content areas generated by participants. During the weekly debriefings, the team discussed the process of the in-depth interviews and assessed saturation. Sampling ceased when thematic redundancy was confirmed. Due to time constraints, transcripts were not returned to participants. However, three participants volunteered for clarification via follow-up emails, which were incorporated.

### Data analysis

Transcripts of the in-depth interviews were auto-generated using Zoom's built-in transcription function; responses in Spanish were translated to English using the Zoom translation function. To ensure the accuracy of the transcription, the interviewers reviewed and compared each transcription to its corresponding audio file. Spanish responses were verified by the bilingual interviewer. Any inaccuracies in the transcription were corrected. Notes taken during and after the in-depth interviews were saved in electronic format and added to the appropriate in-depth interview transcript as interview notes.

Researchers employed a content analysis approach to analyze the in-depth interview data (16, 17). The CDC interviewer used the MAXQDA software package to facilitate data organization and coding. Each transcript file was uploaded into MAXQDA, and a codebook was developed and refined as part of an iterative coding and analysis process. Two coders (DRB, MC) independently coded the first five transcripts, compared codebooks, and resolved discrepancies; thereafter, they coded the remaining transcripts independently with weekly adjudication sessions. A third team member (SSW) audited 20 % of coded files. Themes emerged inductively from patterns in coded segments. An inductive coding tree with hierarchical parent-child nodes was iteratively refined. Coded segments were then mapped deductively to lived-experience experts' recommendations. The research team addressed reliability and controlled for biases by holding regular meetings to review interpretations and analytical decisions. Triangulation across coders, audit trail, reflexive journaling, peer debriefing, and saturation documentation bolstered credibility and dependability.

The analysis explored the influence of lived-experience experts' recommendations on care. To focus on topics lived-experience experts had emphasized in the webinars for care providers, recommendations mentioned more than once by the same lived-experience expert, or by more than one expert, were extracted from webinar recordings. The data were used in aggregate to analyze and identify themes. After the key themes were identified, they were summarized (16, 17). We identified matches and nonmatches between patients' perspectives on their care and lived-experience experts’ recommendations about care, by theme, to explore the influence of the lived-experience experts on care.

The evaluation was deemed a non-research program evaluation by the CDC's human subjects review and was approved by the San Diego State University Review Board (SDSU FWA#00029234), on which FHCSD has IRB reliance. Participating patients completed FHCSD's Broad Consent and authorized the use of their Protected Health Information for research purposes (18). Each participating patient received a US $25 gift card.

## Results

### Patient demographics

Twenty-four patients met the recruitment criteria to participate in the qualitative interviews. Of these, 22 consented and completed interviews, while two declined; their reasons were not recorded. Target saturation (16–24) was achieved after 20 interviews; two further interviews confirmed no new codes. Fifteen interviews were conducted in English and seven were conducted in Spanish. Of the 22 interviewees, 13 were female and nine were male; the mean age was 45.95 years. The mean age by sex shows that females (mean = 43.54) were slightly younger than males (mean = 49.44 years). Eleven of the interviewees were of Hispanic/Latino ethnicity, seven were non-Hispanic, and four were unknown or not reported. Two were identified as multi-racial, 15 as White, and five did not provide information. Nineteen of the patients interviewed identified as heterosexual and three as homosexual. For 12, high school was the highest level of education completed, five had completed less than high school, four completed college or postgraduate education, and one did not provide information. Ten of the patients interviewed were employed, two were students or retired, eight of them were unemployed, and two did not provide information. Eighteen rent their home, three own, and one did not provide information. The distribution of language spoken in the house was the same as the language requested for the interviews—15 speak English and seven speak Spanish. Most (10 patients) had received services at FHCSD for >9 years, six had been FHCSD patients for 3–5 years, and another six for 1–2 years. The top two ways patients learned about this program were through referrals from (1) their primary care provider, as most of the patients were already being seen at FHCSD; and through (2) a health care provider at an urgent care or hospital who was familiar with the Long COVID and Fatiguing Illness Recovery Program at FHCSD.

### Lived-experience experts' recommendations/patients' perspectives on care

Major themes of lived-experience experts’ recommendations to providers included validation of patients' illness experiences; attitudes and beliefs about Long COVID, ME/CFS, and other PAIS; understanding patients' challenges and communicating with empathy; navigating referrals; recognizing and supporting disability; and promoting self-care. In this section of the paper, themes from lived-experience experts' input and patient interview responses are grouped according to these themes.

#### Validation

During educational sessions, lived-experience experts urged providers to validate patients' symptoms and experiences. One of them explained,

“One of the challenges of illnesses like these is that they appear to be invisible. Patients present visually as nondisabled, even though their ADLs (activities of daily living) are significantly impacted”.

Another lived-experience expert elaborated on the theme:

“It's very important to distinguish what you mean by ‘we can’t detect what the problem is’ from actually saying that it doesn’t exist …. reassuring patients that you’re listening to their symptoms and believing them goes a long way” (Webinar on Autonomic/Cardiac Manifestations of Post-COVID Conditions, April 14, 2022).

One of the top four benefits patients reported from their participation was validation of their experience, providing evidence that this message was effectively communicated, internalized, and acted upon by participating providers. Some patients described how the validation of their symptoms helped them be more proactive in seeking help and feel more confident in advocating for themselves.

As the program progressed beyond the first nine months, the lived-experience experts continued to emphasize messages such as “The easiest thing you can do is to validate your patients” (Webinar on patient-led research on post-COVID conditions, August 10, 2023).

#### Attitudes or beliefs about long COVID, ME/CFS, and other PAIS

Lived-experience experts acknowledged the overlap between mental health and chronic disease, and cautioned against assuming symptoms are psychological. One lived-experience expert, a health care provider who has ME/CFS, described that when patients present with a wide variety of symptoms, as patients with Long COVID, ME/CFS, and other PAIS do, medical training may predispose providers to attribute the symptoms to mental illness. The provider explained:

“Understand that sometimes people may … try to hide some of their symptoms because they don’t want to overwhelm you or because they’ve been shut down by other providers in the past, saying, ‘You have a full positive review of symptoms, therefore—so just go see psych’”.

Another lived-experience expert commented:

“What I really want to distinguish is the difference between clinical depression and a depressed mood. If you imagine someone like me, who used to be out in the field working every day, playing sports every other day–now I’m stuck in bed most of the time. I’d be lying if I didn’t say I had a depressed mood, and it would be normal and a rational response to that kind of change. So, this whole distinction between depression and a depressed mood is something I’m hoping providers can really think about and do with a lot of care, before jumping to a diagnosis of depression and prescribing drugs that often exacerbate a lot of the co-occurring conditions, the comorbid conditions we have, like dysautonomia and POTS”.

Almost half of the patients interviewed reported that their attitudes and beliefs about their illness changed because they participated in LC&FIRP. One described this change like this: “I was starting to believe my old doctor. I was like ‘yeah, maybe it's mental. Maybe it's just me and I’m feeling my own symptoms’. The program helped me open up, so it's like ‘okay, so I’m not the only one feeling this way and everything. I am not crazy’”.

Careful navigation of the overlap between Long COVID, ME/CFS, and other PAIS with mental health concerns resurfaced in subsequent webinars throughout the project, in comments like these from lived-experience experts:

“A frequent note of Long COVID clients is that physicians (even some in Long COVID Centers) sometimes attribute their complaints to psychological issues with little or no basis, which then appear in their chart. If the chart entries stress a mental disorder aspect, the patient's physical complaints may not be properly documented or treated”.

“Medical documentation of the physical and cognitive complaints are crucial, as are administering tests to document symptoms and limitations” and

“The suggestion that the Long COVID patient is disabled in whole or part on the basis of a mental health disorder will limit ERISA or Private disability claim payments to two years” (Webinar on Disability and Post-COVID Conditions, November 11, 2022).

#### Understanding patients' challenges and communicating with empathy

Lived-experience experts advised providers to “develop a better, more detailed understanding of the multifaceted issues that patients are facing” and to “communicate with empathy for the toll of the illness on their patients”. Lived-experience experts coached providers to understand with comments like:

“This disease has a tendency to take away from you the person that you were”.

“A lot of us feel like we’re treading water … there's a decrease in daily activities, a sense of hopelessness, decreased social support because very few people understand the disease, and our costs are going up, and ability to earn money—because our work is limited—is going down”.

“Imagine if all your daily activities changed … that's what your patients are facing. Think about if you had to change all your activities and all those things that you worked your entire career to be able to do, you were no longer able to easily do”.

“Many of us … have had a tremendous amount of loss after getting our diagnosis … a loss of identity, a loss of the skills that we had, the brain we used to use, our communications style, the relationships that we've had, the ability to do work or not do work, the ability to do volunteer work or not to do volunteer work, to have a child, to have a pet. These things have a serious impact”.

Lived-experience experts pointed out that patients struggle to find fulfillment given the limitations imposed by their illness. They emphasized that “having an individual plan and having that acknowledged is an important part of healing”. They advised providers to ask about patients’ treatment goals and to encourage patients to “start focusing on enjoying the things they can do rather than dwelling on all the things they can’t do”.

One lived-experience expert, who is also a healthcare provider, emphasized the importance of conveying empathy with messages like, “I’m going to walk with you through this”.

Interviewed patients reported positive interactions with providers in the program: “I feel like I’m finally getting appropriate attention”, “They communicate with me”, and “They are always responsive to my needs”.

Messages from lived-experience experts about communicating with empathy were elaborated as the project continued beyond the first nine months, for example, in comments such as “Communicate that you deeply understand it is a biomedical illness”.

#### Referrals

Lived-experience experts also addressed referrals in their comments during educational sessions with providers. One of them mentioned the need for help navigating the healthcare system, so patients can conserve their energy for attending appointments.

“Providers can support patients by referring them to case management. If case management is not available in the clinic, patients may be able to access it through their insurance. If that's not an option, see if they can get on home- and community-based services through Medicaid”.

Patients did not refer to case management by name, but did express a need for additional assistance from the administration in scheduling and tracking follow-up appointments. Most patients interviewed believed the Long COVID and Fatiguing Illness Recovery Program met their needs in managing their PAIS symptoms. One respondent commented that “personally, I don’t have any complaints about the care they gave me, they helped me a lot. They provided me with everything I needed, such as medicines and any other services. The truth is all the attention was very good”.

Although patients reported that access to specialists was one of the top four benefits of the program, some patients said they were dissatisfied with the (lack of) care, responsiveness, and empathy they received from specialists to whom they were referred outside the program. Among patient interviewees who provided recommendations for program improvement, the most common suggestion was to improve appointment availability for specialists. About a third of respondents mentioned difficulty scheduling appointments and seeing specialists and therapists because of unavailability. Indeed, one respondent commented, “one aspect that I would like to improve would be that there were more appointments and specialists for [people with] severe symptoms. We need more doctors who specialize in these types of patients”. Another respondent commented, “They only really have appointments for physical therapy every other week at most. That hasn’t been helpful, especially because the physical therapists have been saying like ‘if you really want to see any improvement, it will really have to be two to three times a week’”. A few respondents mentioned that administrative staff encouraged them to schedule follow-up appointments immediately because specialist and therapist schedules were often full for weeks to months.

A later webinar in the series expanded on the messages to providers about referrals by emphasizing the referral of patients to specialists familiar with PAIS symptoms, i.e., “so they are properly tested for cognitive deficits, autonomic nervous system issues, cardiac issues, and respiratory issues” (Webinar on Disability and Post-COVID Conditions, November 11, 2022).

#### Recognizing and supporting disability

Lived-experience experts raised awareness about disability associated with Long COVID, ME/CFS, and PAIS, and the services and supports that patients may be eligible to receive. One commented that:

“A lot of people with Long COVID say they’re struggling with work and need help to stay in their job or need time off and other benefits, and they don’t realize a fluctuating, dynamic chronic illness like Long COVID can be considered a disability and therefore entitled to accommodations under the ADA. Disability benefits to take time off might be an option”.

Some patients reported that unmet needs included help with filing for or receiving disability or employment benefits, as well as problems with health insurance coverage.

In later webinars in this series, lived-experience experts continued to educate providers about disability and supports for patients. Specifically, the messages for providers on recognizing disability included: “Disability under the ADA/section 504/section 1,557 is defined as substantially limiting one or more major life activities. This still counts with a fluctuating limitation that comes and goes”. They also encouraged helping patients navigate the available supports and services, such as referring patients to Disability Support & Services, Centers for Independent Living, or Aging and Disability Resource Centers. They described how people with disabilities staff Centers for Independent Living and offer peer support, information and referral, advocacy, and transition support. According to one lived-experience expert, “ … disability supports and services have made the biggest difference in my quality of life” (Webinar on Patient Resources: Disability & Financial Supports, Peer Supports for LC & FI, March 14, 2024).

#### Encouraging and supporting self-care

Lived-experience experts described how self-management, self-monitoring, and pacing had helped them and advised that providers encourage and support self-care. One lived-experience expert who is also a health care provider said,

“The treatment strategy for deconditioning is to ramp up activity … to start pushing people to get back to normal, whereas for people with Long COVID … the treatment regimen is the exact opposite, where [providers] should really be encouraging patients to pace their activity and really be intentional about taking rest”.

Almost all patients who were interviewed reported changes in how they care for themselves due to being in the program. Examples included: exercising, taking health more seriously, eating healthier—eliminating bad foods and alcohol, wearing masks, avoiding crowds, and using a Fitbit to monitor health and sleep. To underscore these examples, one respondent reported, “I like this program because it helped me to take care of myself, have healthier habits, and know how to identify when my body is getting sick”.

These messages about providers' role in supporting patients' self-care were reinforced in later webinars, e.g.,

“Self-management is the patient's day-to-day management of their chronic health condition. The goal of self-management is increasing the patients' involvement in and control over their care. Better self-management equals improved symptom management and quality of life”.

“Self-monitoring offers valuable data for patients and providers”.

“The benefits of self-management are the strategies are portable, allow patients to be an active participant in their care, [and] help patients recognize the power they have in managing their own condition; don’t assume that patients know about self-management” (Webinar on Practical Strategies for Symptom Management, July 14, 2022).

#### Unmatched comments

Patients highlighted one issue not mentioned by lived-experience experts: the need for printed resources and brochures in Spanish. Likewise, one theme of lived-experience experts' input, the importance of providers acknowledging their uncertainty given the limited evidence base to support care, did not come up in patient interviews. On this topic, one lived-experience expert advised, “With a disease like this where the data is emerging rapidly and it's not quite clear, it's important to have humility”. Other lived-experience experts encouraged providers to be comfortable with uncertainty, with comments like “Being able to admit you don’t know everything … that's OK … these patients understand that”.

## Discussion

By including people with lived-experience of Long COVID, ME/CFS, and other PAIS as subject matter experts for LC&FIRP medical education activities, we aimed to foster empathy and responsiveness to patients' problems as they experience them, not only as professionals define them. Qualitative evaluation results reported herein suggest at least partial achievement of this aim. Patients' perceived benefits of LC&FIRP included effective communication with and responsiveness from providers, as well as validation of patients' illness experiences, aspects of the therapeutic relationship that were highlighted by lived-experience experts who provided recommendations during training for providers. The positive impact was underscored by patients' reports that providers' belief in their symptoms encouraged positive shifts in their self-care.

Validation of illness experience is an important aspect of the relationship between patients and providers that has proven challenging for those with PAIS, like ME/CFS, that involve medically unexplained physical symptoms (19). These health conditions have characteristics that make them less recognizable and more challenging to treat, i.e., not being organ-specific, and not having objective diagnostic signs and or widely accepted therapeutic options; for these reasons they have been described as “neglected”, “poorly understood”, and “invisible” (20–23). This is especially true for those with the most severe symptoms, who may be bedbound or housebound. Inclusion of caregivers and patient advocates as lived-experience experts in LC&FIRP medical education activities helped to represent those most severely affected by Long COVID, who were themselves unable to participate.

Qualitative research into the lived experience of ME/CFS has highlighted how the dominant illness model in medical education can obstruct the relationship between health care providers and patients with these illnesses (24). As Bayliss et al. (25) conclude, “the biomedical approach, which is central to the medical curriculum, leads many health care professionals to conclude that there is no real illness [in ME/CFS], as there is currently no identifiable pathology”. Patients' negative interactions with a healthcare system that doesn't recognize their illness experience add to what are already daunting challenges imposed by the illness (22, 26–29). When physical symptoms cannot be detected or explained by current biomedical knowledge and technology, good provider-patient partnerships are crucial (30). Our interviews of patients who received care from LC&FIRP participating providers suggested that patients perceived providers as understanding of patients' illness experiences, communicating with empathy, and encouraging and supporting their self-care. The experience with LC&FIRP is consistent with previous research indicating that involving people who have accessed health care services and systems is an educational strategy that nurtures the development of provider skills, knowledge, and attitudes essential for effective medical practice (31–33). Other ECHO projects, e.g., the Missouri Show ME Autism ECHO project, have introduced lived-experience experts into their medical education activities and report the benefits of this role (34).

Stigma and inequities in access to care associated with invisible illnesses like PAIS can transcend sociodemographic hierarchies (34). In any clinical setting, dismissal, disbelief, and denial by providers not equipped with the knowledge and therapeutic skills required to diagnose and manage these conditions can deprive people with PAIS of recognition, support, and services (27). When the dismissal of concerns and symptoms by service providers and employers is compounded by demographic, ethnic, social, and economic pre-existing structural disparities, the invisibility and inequities are exacerbated (27, 35, 36). For this reason, listening to patients is especially important in clinical settings like the Federally Qualified Health Center network, where LC&FIRP was implemented, in which the majority of patients are people of color, 91% have low income, and 29% are uninsured (37, 38).

What patients said unprompted in interviews indicated that providers were addressing some themes of lived-experience experts' input, notably their recommendations to validate patients' illness experience, understand patient challenges, communicate with empathy, and support self-care. However, patients also pointed to other themes lived-experience experts emphasized in trainings that patients perceived were not being addressed in the health care setting. These included challenges with referrals to specialists and help filing for or receiving disability or employment benefits, and problems with health insurance coverage, which patients mentioned were unmet needs. These are challenges that affect people with a wide variety of health problems, that involve not only provider practice, but broader health system organization features, and are unlikely to be solvable through medical education alone. Some topics that lived-experience experts raised for providers to consider were not reflected in what patients said in interviews, most notably, the importance of providers acknowledging their uncertainty, given that knowledge about Long COVID, ME/CFS, and other PAIS is evolving. The evidence base for managing and treating symptoms is limited. That patients didn't raise this issue could mean that providers did not address the recommendation or that they did address it, but that patients didn't notice this or chose not to mention it.

The inclusion of lived-experience experts who are themselves health care providers, in addition to patients who are involved with patient advocacy, is a strength of LC&FIRP. Pairing these lived-experience experts with specialists in various medical disciplines who care for patients with Long COVID, ME/CFS, and other PAIS provided a wide diversity of perspectives in the LC&FIRP application of Project ECHO's “All Teach. All Learn” tele-mentoring model (39). As for a qualitative evaluation of patients' experiences of application of the ECHO model to chronic pain management in the primary care setting, findings from this evaluation of LC&FIRP suggest that benefits to patients are positive and significant, if indirect (40).

## Limitations

This qualitative evaluation had some design limitations. The included recommendations of the lived-experience experts are illustrative of what some lived-experience experts want providers to address in their health care practice related to Long COVID, ME/CFS, and other PAIS. Likewise, the comments of interviewed patients illustrate perceptions of some patients in the LC&FIRP program. Representativeness of neither the lived-experience experts' recommendations nor the patients' perspectives is assured. Offering a gift card to patients may have favored participation by those with greater financial need, although that seems unlikely, given that 91% of patients at FHCSD have low income. The inclusion of five lived experience experts who are affiliated with patient advocacy organizations may have resulted in recommendations that underrepresented the lived experience of patients who are not as active or empowered. The two health care providers included as lived-experience experts may have provided recommendations that other patients would not have provided given their knowledge about health care services.

Since we included only recommendations of lived-experience experts that were made more than once, to ensure focus on emphasized points, we may have excluded some important input.

This evaluation covered the first nine months of the program and aimed to identify necessary adjustments for the future. Ideally, we would have interviewed providers and LC&FIRP faculty as well as patients. We did not evaluate changes in providers' practice after exposure to LC&FIRP training, as that evaluation is planned at the end of the project. We included lived-experience recommendations from webinars, but not the weekly tele-mentoring sessions, to protect information from these case consultation discussions. Nonetheless, given that many of the same lived-experience experts participated in both weekly sessions and the webinars, we assumed they provided the same or similar messages. We also thought that all providers who participated in the program were exposed to all the major messages of the lived-experience experts we highlight in this manuscript, because the same lived-experience experts participated in both weekly sessions and webinars, and messages were repeated.

Medical experts in the training sessions echoed some recommendations of lived-experience experts; therefore, we are unable to attribute provider uptake of these recommendations solely to the influence of lived-experience experts. Patient interview questions did not specifically ask about the themes brought up by lived-experience experts in the educational sessions with providers. Instead, we highlighted patient comments related to these themes that were made spontaneously in response to more general questions. While this approach elicited responses from patients that were top of mind for them, patient perspectives on topics the patients did not bring up remained unexplored.

## Conclusion

The LC&FIRP is based on the Extension for Community Healthcare Outcomes (ECHO) model, a case-based virtual community of practice learning framework developed by the University of New Mexico designed to disseminate and implement best practices, especially in under-resourced areas. LC&FIRP integrated lived-experience experts, who joined faculty from multiple medical disciplines, in mentoring the participating clinicians on providing care for their PAIS patients. Positive comments from patients about topics emphasized by the lived-experience experts provide evidence of providers' uptake and application of their recommendations, supporting the value of involving lived-experience experts in medical education to improve health services. Findings from this evaluation may have implications for medical education about other complex health conditions. Investigation of the perspectives of practicing health care providers on the inclusion of lived-experience experts in continuing medical education could be a productive area for future research.

## Data Availability

The raw data supporting the conclusions of this article will be made available by the authors, without undue reservation.
