# Supplementary material for: Assessing the influence of lived-experience experts on healthcare providers in a virtual community of practice: a qualitative study
Source: Front Health Serv. 2025 Jun 27;5:1562651. doi: 10.3389/frhs.2025.1562651 (PMC12245761; doi:10.3389/frhs.2025.1562651)
Supplement: Supplementary file 2 [file Datasheet2.pdf]

Supplemental Table 2: Interview Guide

Evaluation Component	Dimension (Question Intent)	Questions asked of participants
Demographics	Information collected on the following to appropriately describe the participant sample.	1. Sex<br>2. Age<br>3. Ethnicity and race<br>4. Sexual Orientation<br>5. Education<br>6. Employment<br>7. Housing—Own/Rent<br>8. Language spoken at home<br>9. Veteran status<br>10. How Long have they been treated at FHCSD
COVID History		1. Have you ever experienced Long COVID, ME/CFS, or other post-infectious fatiguing illness symptoms?<br>2. Are you still experiencing symptoms?<br>3. How Long (did you)/(have you been) experiencing symptoms?
Introduction		1. To get us started, tell me about your experience with Long COVID & Fatiguing Illnesses Recovery Program study.
Program Assessment		
Program	Benefit—"To assess participants perceived benefit from participating in the LC&FIRP."	1. What do you find beneficial about participating in this study?
	Unmet Needs—"To assess how well program content meets program participants' needs; additionally, to evaluate if there are content gaps."	2. How well are your needs as a person experiencing Long COVID/ME/CFS/fatiguing illness syndrome being managed?

		3. What other needs do you have that have not been addressed?
Overall	Improvement—"To assess the way participants think the program can be improved."	4. How could the care you received be improved?
Post-Intervention Effects		
Capacity/Accessing Care	Changes in Ability/Confidence/Skill—"To assess changes in participants' perceived ability, confidence or skill because of program participation."	5. How well are you able to get the healthcare you need as a person living with Long COVID/fatiguing illness?<br>6. What makes you feel this way?
Practice/Changes in Self-Care	Changes in Knowledge, Attitudes, Behaviors and (individual) practice—"To assess changes in participants' perceived knowledge, attitudes, behaviors or practice because of program participation	7. How have you changed how you take care of yourself as a person living with Long COVID/fatiguing illness?<br>8. How has being in the study impacted your attitudes or beliefs about your symptoms?
Closing		9. Is there anything else you would like to share about your experience participating in the Long COVID & Fatiguing Illnesses Recovery Program?
